# On the Image Sensor Processing for Lane Detection and Control in Vehicle Lane Keeping Systems

**DOI:** 10.3390/s19071665

**Published:** 2019-04-08

**Authors:** C.Y. Kuo, Y.R. Lu, S.M. Yang

**Affiliations:** International Program on Energy Engineering, National Cheng Kung University, Tainan 70101, Taiwan; river85511@gmail.com (C.Y.K.); yoyo21308@gmail.com (Y.R.L.)

**Keywords:** image sensor, lane detection, lane control

## Abstract

Lane keeping systems for a keeping a vehicle in the desired lane is key to advanced driving assistance system in autonomous vehicles. This paper presents a cost-effective image sensor with efficient processing algorithm for lane detection and lane control applications to autonomous delivery systems. The algorithm includes (1) lane detection by inverse perspective mapping and random sample consensus parabola fitting and (2) lane control by pure pursuit steering controller and classical proportional integral speed controller based on a nonholonomic kinematic model. The image sensor experiments conducted on a 1/10 scale model car maneuvering in a straight–curve–straight lane validate the better processing performance before, during, and after the turning section over previous work. The image sensor with the processing algorithm achieves the average lane detection error within 5% and maximum cross-track error within 9% in real-time. The development shall pave the way to cost-effective autonomous delivery systems.

## 1. Introduction

It is known that the vast majority of all reported road accidents are due to human faults [1]. Advanced Driving Assistance System (ADAS) has recently been proposed to predict driver’s intent, warn the driver about possible lane departure, and assist lane keeping [2], and it has been implemented on several vehicles in automobile industry. The reference path and the vehicle position can be determined from the maps created by geographic information system (GIS); however, the path and position accuracy often suffers from map resolution, data availability, and real-time update limitations. An on-board vision-based lane keeping system is therefore necessary to generate a reference path, obtain accurate vehicle position relative to the lane, and control the vehicle safely in a desired path.

A recent review summarized most vision-based lane detection methods that share three common steps [3]: (1) bird’s eye view transformation by inverse perspective mapping [4,5], (2) lane feature detection by edge-based [6,7,8] or color-based methods [9], and (3) lane fitting by random sample consensus (RANSAC) combined with least-square line [10], B-spline [11], or hyperbola pair fitting [12] methods. Edge-based methods only rely on intensity information, thus require less computation for real-time applications, which proved to be key to cost-effective, autonomous vehicles in goods delivery. Lane fitting by the random sample consensus combined with parabola pair is considered the most applicable. Lane controllers can be designed based on nonholonomic kinematic or dynamic models. A proportional–integral–derivative (PID) steering controller [13] and a pure pursuit steering controller [14,15] have been proposed to determine vehicle steering angle to maintain safe driving in a lane. The above studies require on-board LIDAR (Light Detection and Ranging) with heavy computation load for lane keeping and radar for adaptive lane control.

In addition to chauffeuring people, another major function for autonomous vehicles is the delivery of goods. It is expected that 80% of all packages will be delivered autonomously in the next decade [16].

The world’s e-commerce, in particular in USA and China, has doubled in the past 10 years and is expected to double again in the next five [17]. A cost-effective lane keeping system is therefore key to the development of autonomous delivery systems. This work proposes a lane keeping system for both lane detection and lane control by using only a low-cost image sensor (dashcam) with an efficient processing algorithm for real-time applications. In lane detection, inverse perspective mapping (IPM) followed by edge-based detection and RANSAC parabola fitting are applied to obtain accurate vehicle position relative to the lane center. In lane control, both pure pursuit steering controller based on a nonholonomic kinematic model and proportional-integral speed controller are adopted to maintain the vehicle safely in a desired lane. The image sensor with the processing algorithm is shown to be effective for both lane detection and lane control. The on-board processing time of the algorithm is more efficient than that of the previous work. With the advent of unmanned autonomous vehicles in the delivery market, this work may be one of the latest steps for applications to lower delivery costs of everyday items.

## 2. Lane Detection by Image Sensor

**Figure 1:**
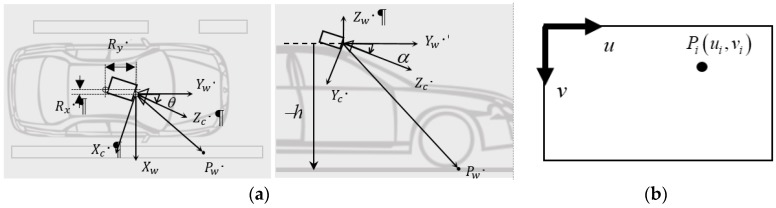
(**a**) An image sensor (dashcam) with the camera frame $\left(X_{c},Y_{c},Z_{c}\right)$ mounted on a vehicle at height *h* above ground with pitch angle α, yaw angle θ, and offset $\left(R_{x},R_{y}\right)$ from the vehicle center in the world frame $\left(X_{w},Y_{w},Z_{w}\right)$, and (**b**) illustration of the image sensor plane, where point $P_{i}$ is the projection on the image plane of a point $P_{w}$ on the ground.

The vision-based lane detection is in three common steps: (1) image transformation from the sensor’s frontal view to bird’s eye view, (2) edge-based lane feature detection, and (3) lane markers regeneration or reconstruction in the processing image. In lane detection, the sensor image from a dashcam first has to be transformed from frontal view into bird’s eye view so that the lane markings on become parallel (assuming constant lane width) for accurate vehicle positioning. Consider the image sensor with camera frame $\left(X_{c},Y_{c},Z_{c}\right)$ mounted with pitch angle α, yaw angle θ, offset $\left(R_{x},R_{y}\right)$ on a vehicle in the world frame $\left(X_{w},Y_{w},Z_{w}\right)$ at height *h* above the ground, as shown in Figure 1a. The transformation from an arbitrary point $P_{w}\left(x_{w},y_{w},-h\right)$ in the world frame to the corresponding point $P_{i}\left(u_{i},v_{i}\right)$ in the image plane as shown in Figure 1b can be determined by coordinate transformation:

(1) $$u_{i}=c_{u}+f_{u}\;\left(x_{w}cos\theta -y_{w}sin\theta \right)/d$$

(2) $$v_{i}=c_{v}+f_{v}\;\left(-x_{w}\;sin\alpha \;sin\theta -y_{w}\;sin\alpha \;cos\theta +h\;cos\alpha \right)/d$$

where $\left(f_{u},f_{v}\right)$ and $\left(c_{u},c_{v}\right)$ are the focal length and the image sensor’s optical center, respectively, and $d=x_{w}\;cos\alpha \;sin\theta +y_{w}\;cos\alpha \;cos\theta +h\;sin\alpha .$ The inverse perspective mapping from the image plane to the ground plane can be obtained by

(3) $$x_{w}=-h\left(-\left(\left(u_{i}-c_{u}\right)/f_{u}\right)cos\theta +sin\theta \left(\left(\left(v_{i}-c_{v}\right)/f_{v}\right)sin\alpha -cos\alpha \right)\right)/e$$

(4) $$y_{w}=-h\left(\left(\left(u_{i}-c_{u}\right)/f_{u}\right)sin\theta +cos\theta \left(\left(\left(v_{i}-c_{v}\right)/f_{v}\right)sin\alpha -cos\alpha \right)\right)/e$$

where $e=\left(\left(v_{i}-c_{v}\right)/f_{v}\;\right)cos\alpha +sin\alpha$.

**Figure 2:**
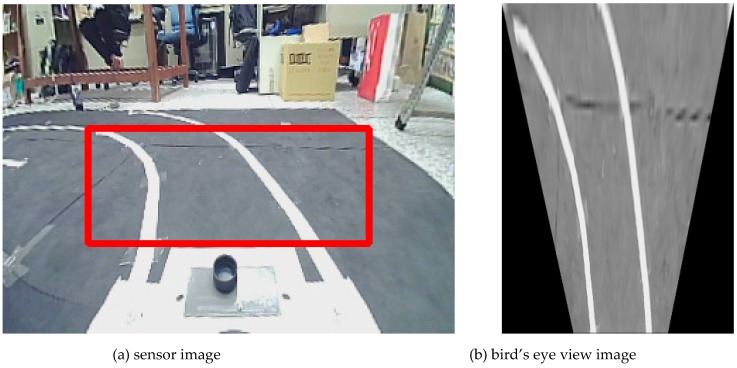
Illustration of the inverse perspective mapping (IPM) by converting (**a**) the region of interest (in box) in the sensor image to (**b**) the bird’s eye view image for detecting the parallel lane markings.

**Figure 3:**
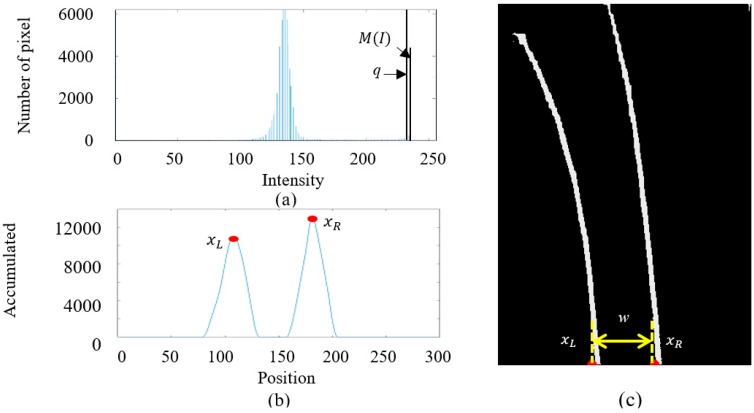
(**a**) Histogram of the grayscale bird’s eye view image with M(I) representing the highest intensity value and the intensity threshold *q*, (**b**) the column intensity sum of the bottom half of the image with peaks at the left and right lane markings, $x_{L}$ and $x_{R}$, and (**c**) the result of thresholding with lane width *w* and position of $x_{L}$ and $x_{R}$.

Figure 2a illustrates the inverse perspective mapping where the sensor image is transformed by inverse perspective mapping (IPM) into a bird’s eye view image. After IPM, the lane features such as intensity and geometry of the lane markings are preserved as shown in Figure 2b, and they can be applied to locate the lane position by edge-based lane feature detection. The intensity difference between the lane markings and the ground pavement is often so strong that the IPM image can be converted to binary grayscale image and then filtered by an intensity threshold *q*:

(5) q=kM(I)

where *k* is a constant to preserve lane markings, M(I) is the peak with the highest intensity value in the histogram of the grayscale image, and I is the grayscale image. Figure 3a illustrates the histogram of the gray scale IPM image. The column intensity sum of the binary image can then be applied to locate the horizontal position $x_{L}$ and $x_{R}$ of the left and right lane marking and the lane width *w*, as shown in Figure 3b,c.

**Figure 4:**
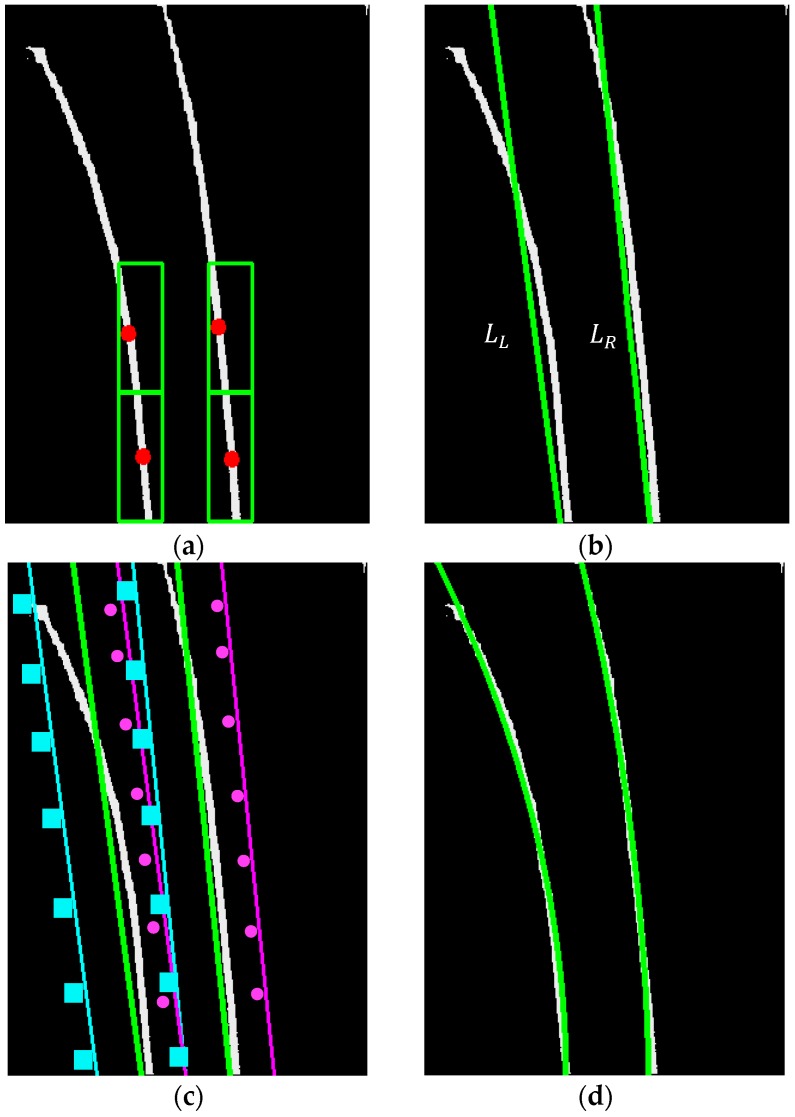
Illustration of (**a**) lane detection for the slope of the line segments $L_{L}$ and $L_{R}$ by the center points of the two windows at the left/right lane markings and (**b**) lane detection for line segments $L_{L}$ and $L_{R}$ from the base to the lane markings. Illustration of curve lane fitting by (**c**) the square lines for $L_{L}$ and the dotted lines for $L_{R}$ (**d**) by using the RANSAC parabola fitting.

Note that the lane markings close to the vehicle are approximately vertical after the transformation, thus the horizontal position of the left and right lane marking can be determined by the column intensity sum of the threshold image, and two lines $L_{L}$ and $L_{R}$ passing through $x_{L}$ and $x_{R}$, respectively, are the initial guess of the position of the left and right lane marking. Their slope can be determined by the two windows of width w/2 and height *b* at horizontal position as shown in Figure 4a. By the intensity center of each of the two windows, the slope of $L_{L}$ and $L_{R}$ can be determined as indicated in Figure 4b.

One of the major challenges in autonomous delivery vehicle is lane detection when making sharp turn. In order to obtain a better position estimate in curve lane markings, random sample consensus (RANSAC) parabola fitting is applied around $L_{L}$ and $L_{R}$ for lane feature detection. The parabola fitting is to divide each window into sections equally spaced in the y-direction, as shown in Figure 4c, select randomly one point from each section to define parabola geometry by the least square method, and calculate the accumulated intensity value of the parabola. By use of the central limit theorem, a parabola can be estimated accurately as shown in Figure 4d. The highest accumulated intensity value in each window can be used to locate the position of each lane markings. Accurate vehicle position with respect to the lane center is necessary for lane control to minimize the cross-track error and keep the vehicle driving safely in the desired lane. The model assumes the lane markings to be parallel is applied to obtain the lane center position. The accurate vehicle position relative to the lane center can then be easily obtained by considering the offset $\left(R_{x},R_{y}\right)$ of the image sensor from the vehicle center.

## 3. Lane Control by Image Sensor

**Figure 5:**
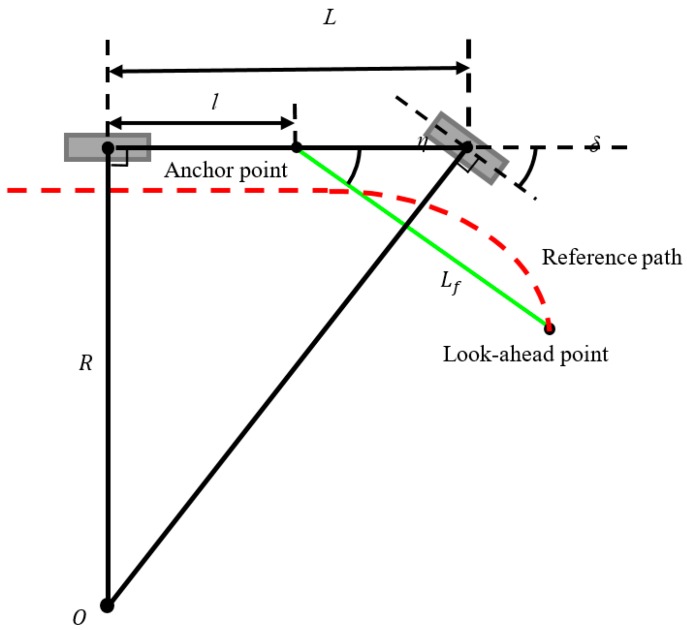
Illustration of lane control on a curvature of radius *R* with geometric description between the steering angle δ and wheel base *L*, the distance of the forward anchor point l, forward drive look-ahead distance $L_{f}$, and heading of the look-ahead point from the forward anchor point to the vehicle heading η.

A pure pursuit steering controller and a PI speed controller were applied to keep the vehicle driving safely along the detected lane center at a desired velocity. The former controller calculates a kinematically feasible path for vehicle to maneuver from its current position to goal position. This is used for most vehicles with no universal wheels installed. The latter controller allows the vehicle to follow the calculated curve path, and classical PI control was adopted for acceptable control performance with low or no computation loading. The graphical description of a pure pursuit controller based on a nonholonomic kinematic model [18] is shown in Figure 5, where *L* is the vehicle wheelbase, *l* is the distance from the rear axle to the forward anchor point defined as the center of the vehicle, $L_{f}$ is the forward drive look-ahead distance, and η is the heading of the look-ahead point (constrained to the reference path) from the forward anchor point with respect to the vehicle heading. In steering control, the steering angle δ can be determined by $\delta ={tan}^{-1}(L/R)$, where *R* is the distance from the instantaneous rotation center *O* to the rear axle, $R=\left(L_{f}/2+lcos(\eta \right))/sin\left(\eta \right)$. The forward drive look-ahead distance $L_{f}$ is dependent on the command velocity for stability. Under high velocity conditions, larger $L_{f}$ is required in order to maintain the system stability and determine a more feasible δ for the nonholonomic kinematic model. In speed control, a classical PI controller was adopted without the derivative (D) term for closed loop system stability:

(6) $$u=K_{p}\left(r-s\right)+K_{i}\int_{0}^{t}\left(r-s\right)d\tau$$

where *u* is the nondimensional speed control signal, $K_{p}$ and $K_{i}$ are the proportional and integral gains, respectively, r is the command velocity, and s is the vehicle velocity. The time step dτ≅Δt is identified according to the update rate of the state of the vehicle and the computational speed. The proportional and integral gains $K_{p}$ and $K_{i}$ can be determined by extensive testing guided by the parameter space approach of robust control. By applying the parameter space approach, an area in the $K_{p}-K_{i}$ plane can be determined for which the desired design specifications such as stability, phase margin limitation, and robustness are satisfied. Through actual experiment, the PI controller parameters best fit all the design specifications are then determined.

## 4. Experimental Verification

**Figure 6:**
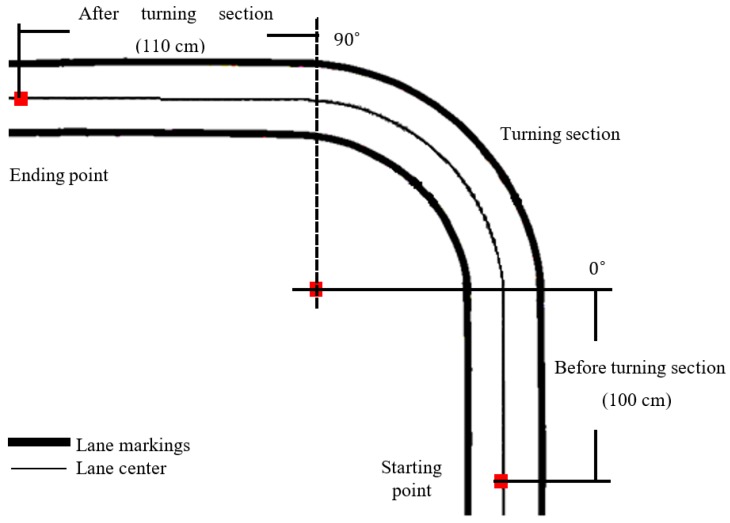
The route with lane center (solid line) and lane markings (thick line) for experimental verification of the image sensor in lane detection and control.

The performance of the image sensor processing algorithm in lane keeping system is verified by a 1/10 scale model car of length 40 cm and width 18 cm maneuvering in straight–curve–straight lane as shown in Figure 6. The car is equipped with an image sensor (fisheye dashcam), inertial measurement unit, and on-board computer (ARM, 2 GHz). The trajectory of the car during experiment is captured by an overhead camera with $90^{{}^{\circ}}$ field of view (FOV), 24 frames per second (fps), and 800 × 600 pixel resolution to observe the cross-track error.

In the experiment, an image sensor with $140^{{}^{\circ}}$ field of view (FOV), 30 frames per second (fps), and 320 × 240 pixel resolution is mounted on the car at height h=21.3 cm above the ground plane; yaw angle $\theta =0^{{}^{\circ}}$, pitch angle $\alpha =20^{{}^{\circ}}$, and offset $\left(R_{x},R_{y}\right)=\left(0,13.5\right)$ cm from the vehicle center. After camera calibration, the focal length $\left(f_{u},f_{v}\right)$ is (189.926, 256.917) pixels, while the camera optical center $\left(c_{u},c_{v}\right)$ is (160.717, 120.688) pixels. Most lane markings belong to a region of interest (ROI) of 260 pixel width and 85 pixel height with the position of the top left corner (30, 90) in the image sensor, as shown in Figure 2a. The image in ROI is then transformed to the bird’s eye view image (300 × 400 pixel) and converted to grayscale as shown in Figure 2b. The intensity histogram of the grayscale IPM image is calculated with the peak marking the highest intensity M(I) as shown in Figure 3a. According to the testing results, k=0.9 is adopted to obtain the best filtering performance by Equation (5). The column intensity sum of the binary image can then be applied to locate the horizontal position $x_{L}$ and $x_{R}$ of the left and right lane marking and the lane width w as shown in Figure 3b,c. With the two windows of width w/2 and height 60 pixels at the bottom of each of the two lane markings as shown in Figure 4a, the slope of each lane marking can be determined as shown in Figure 4b. These slopes are applied to obtain two windows of equal width for spline fitting as shown in Figure 4c. Each window is divided into 10 sections and the number of iterations of the spline fitting is set of 30 to obtain the best performance on the on-board computer, as shown in Figure 4d. Based on the parallel lane model, the lane center can therefore be obtained.

**Figure 7:**
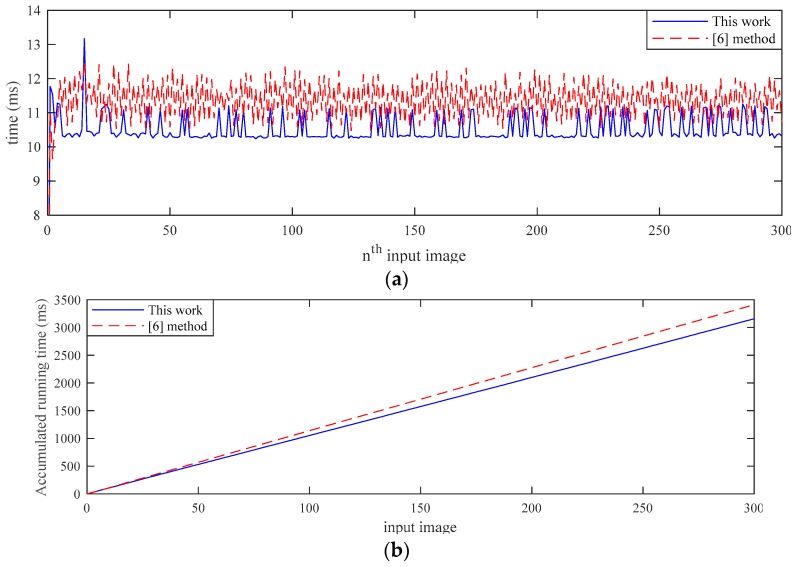
(**a**) Comparison of the processing time in lane detection for a series of 300 images and (**b**) the accumulated computation time validates that the algorithm in this work is more efficient.

The efficiency of the lane detection algorithm is shown in Figure 7a. In a series of 300 captured images of a typical maneuver in a straight lane, the computation time in lane detection by the on-board CPU (2 GHz) is in the range of 10.4 to 11.2 ms. By comparison, the computation time by using the method in [6] is 11.5 to 12.2 ms. The algorithm in this work is shown more efficient in all of the 300 images in lane detection. In addition, the algorithm remains applicable to curve lane, while the method in [6] is otherwise for its limitation when using Hough transform. Figure 7b illustrates the accumulated time of processing the 300 images during vehicle motion of ~10 s. The algorithm is shown to shave 0.5 s of computation time in lane detection, and such saving is critical to the development of autonomous delivery systems.

The lane control experiment is also performed on the 1/10 scale model car. For the pure pursuit controller, the vehicle wheel base is L=26 cm and the distance from the rear axle to the forward anchor point is l=6 cm. The forward drive look-ahead distance $L_{f}$ is dependent upon the command velocity *r* to overcome the stability issue. According to the characteristics of the nonholonomic kinematic model, the relationship between $L_{f}$ and *r* in the experiment is

$$L_{f}\left(r\right)=\{\begin{array}{c} 55\;\mathrm{cm}\;\\ 43\;\mathrm{cm}\;\\ 65\;\mathrm{cm}\; \end{array}\begin{array}{l} \mathrm{if}\;r<1.35\;\left(m/s\right)\\ \mathrm{if}\;1.35\;\left(m/s\right)<r<1.5\;\left(m/s\right)\\ \mathrm{otherwise}. \end{array}$$

For speed control, the PI controller parameters $K_{p}$ and $K_{i}$ are determined through the parameter space by experiment: $K_{p}=0.3$ and $K_{i}=0.04$. For the integration, the time step is set at 5 ms to achieve the best performance.

**Figure 8:**
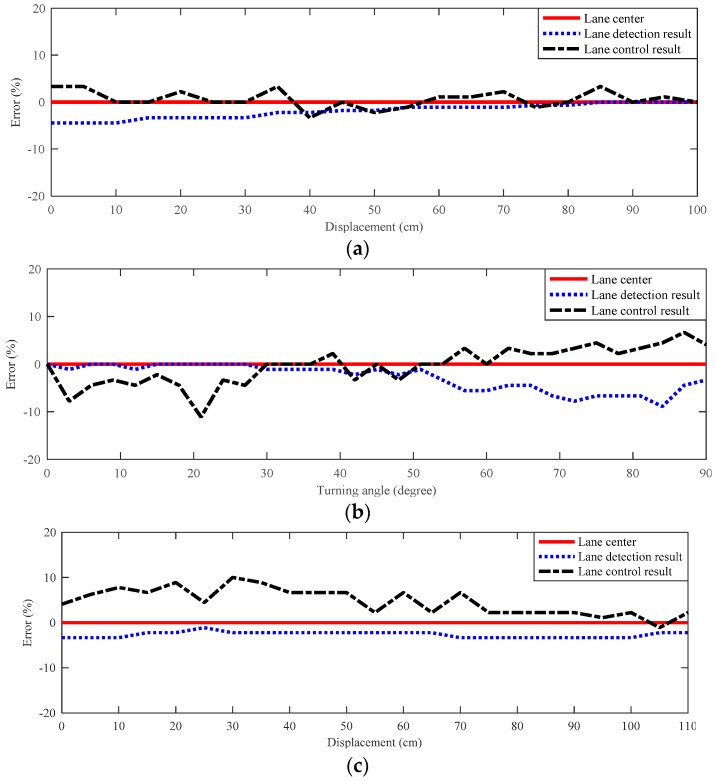
Error percentage in the experimental results of the image sensor in lane keeping system (**a**) before, (**b**) during, and (**c**) after the turning section, where the error percentage is defined as the ratio of the offset from the lane center to the lane width.

The experiments were conducted with the model car maneuvering at a speed of 1 m/s in a straight–curve–straight lane (radius 99 cm) and lane width 37 cm. The vehicle speed is set at high enough to generate ~1G centrifugal acceleration during the turning section to simulate real-world vehicle operation. The experiment results for lane detection and lane control are shown in Figure 8, where the solid line represents the real lane center, the dotted line the lane control results, and the dash line the lane detection result. The error percentage is defined by the ratio of deviation over the lane width. Since the width of the lane markings in the experiment is 35 cm, the lane detection error is expected to be within ±1.75 cm (±5%). As for cross-track error, the maximum tolerable cross-track error is ±9.5 cm (±25%), which is the maximum offset of the vehicle from the lane center without crossing the lane markings. The average lane detection error and maximum cross-track error in these three sections are 2.54%, 3.37%, 4.41%, and −4.44%, −8.89%, −4.08%, respectively. Due to the small steering angle ($3^{{}^{\circ}}-5^{{}^{\circ}}$) adjustment is not possible, and there is a slight increase during the turning section. Even if the lane keeping system is capable of detecting the small offset from the lane center and sending a corresponding command to the steering angle during the turning section, the car is still not capable of adjusting its steering angle due to backlash in hardware constraint. However, even under this limitation, the performance of the vision-based lane keeping system can still be clearly indicated by the maximum cross-track error in the experiment within 9%.

## 5. Conclusions

(1) A cost-effective lane keeping system is key to the development of autonomous delivery systems. This work proposes a lane keeping system for both lane detection and lane control by using only a low-cost image sensor (dashcam) with an efficient processing algorithm for real-time applications. A vehicle with only an image sensor, without LIDAR or radar, is shown to be capable of lane detection and lane control with good accuracy in real-time. The image processing algorithm in lane detection includes (a) inverse perspective mapping to transform the dashcam image to bird’s eye view image, (b) binary and histogram filter to detect the lane slope feature, and (c) random sample consensus parabola fitting to reconstruct the lane markings.
(2) The performance of the image sensor processing algorithm in lane keeping system is verified by a 1/10 scale model car of length 40 cm and width 18 with an image sensor (fisheye dashcam), inertial measurement unit, and on-board computer (ARM, 2GHz). The efficiency of the lane detection algorithm is ~11 ms for lane detection of a typical image, which is ~5–10% lower than that in comparable study [6]. The algorithm in this work is shown more efficient in all of the 300 images in lane detection. In addition, the algorithm remains applicable to curve lane. In lane control, the trajectory of the car during experiment is captured by an overhead camera to observe the cross-track error. Experiments conducted on the model car maneuvering at speed 1 m/s in a curve lane of radius 99 cm for ~G acceleration further show that that the average lane detection error before, during, and after curve lane are all within 5%. The image sensor with the processing algorithm is effective in advanced driving assistance of autonomous delivery systems. In lane control, a pure pursuit steering controller and a proportional integral speed controller based on a nonholonomic kinematic model are applied to show that the maximum cross-track error is within 9% in lane control. Both the lane detection and lane control performance are critical to the development of autonomous delivery systems.
(3) The proposed lane keeping system is considered effective and efficient. Future development will simulate more complicated road conditions such as rugged ground and light conditions, such as glare when facing the sun and dim light when driving in a tunnel. Rugged ground may cause the vehicle to tilt, thus changing the yaw and pitch angle of the vehicle, and affecting the accuracy of inverse perspective mapping. Glare and dim light conditions may reduce the intensity difference between the lane markings and the road pavement, so that detecting lane features using the edge-based method may therefore be challenging.
